# Muscularis propria-level endoscopic submucosal dissection for a primary rectal mucosa-associated lymphoid tissue lymphoma

**DOI:** 10.1055/a-2988-7853

**Published:** 2026-11-03

**Authors:** Sandro M Hasenhütl, Kristijan Skok, Azab M. El-Shabrawi, Saulius Mikalauskas, Gregor Gorkiewicz, Robert Sucher, Mohamed El-Mahrouk

**Affiliations:** 1 Division of General, Visceral and Transplantation Surgery, Department of Surgery 31475 Medical University of GrazGrazAustria; 2 Diagnostic and Research Institute of Pathology 31475 Medical University of GrazGrazAustria

A 51-year-old man underwent screening colonoscopy. A broad-based 30-mm polypoid lesion was found in the mid rectum, 10 cm from the anal verge. Narrow-band imaging suggested JNET type 3, prompting biopsy. Histology and immunophenotyping demonstrated extranodal marginal zone lymphoma of mucosa-associated lymphoid tissue (MALT lymphoma). Positron emission tomography/computed tomography, gastroscopy with mapping biopsies, bone marrow biopsy and pelvic magnetic resonance imaging showed no further manifestation (Lugano stage I; low-risk MALT-IPI). 1​ Following the multidisciplinary discussion of radiotherapy versus local resection, endoscopic submucosal dissection (ESD) was selected with curative intent. Although radiotherapy is established, 1​ complete endoscopic resection with observation has shown favourable outcomes, predominantly for smaller lesions. 2​ 3​ ​4​

ESD was performed under general anaesthesia using a HybridKnife Flex (Erbe Elektromedizin, Tübingen, Germany; Video 1 ). Injection produced poor lifting over previous biopsy sites and minimal central lift. The submucosa was strikingly dense and whitish, preventing reliable distinction between biopsy-related fibrosis and possible lymphomatous extension and obscuring a consistent dissection plane. To maximize the likelihood of a negative deep resection margin while maintaining orientation, dissection was continued immediately above the muscularis propria, which provided a continuous landmark. En bloc resection was achieved. A 1-mm muscularis propria defect was closed with two clips. The course was uneventful.

**Video 1** Endoscopic submucosal dissection of a primary rectal MALT lymphoma immediately above the muscularis propria, as dense biopsy-related fibrosis was indistinguishable from possible lymphomatous extension and obscured the submucosal plane.


Histology confirmed mucosa-confined MALT lymphoma with free deep and lateral margins (R0; Fig. 1 ). Thus, retrospectively, the deep plane was not required oncologically. Eight days after ESD, the multidisciplinary lymphoma board recommended surveillance without additional treatment, with endoscopy at 3 and 6 months and then every 6 months for 2 years.

**Fig. 1:**
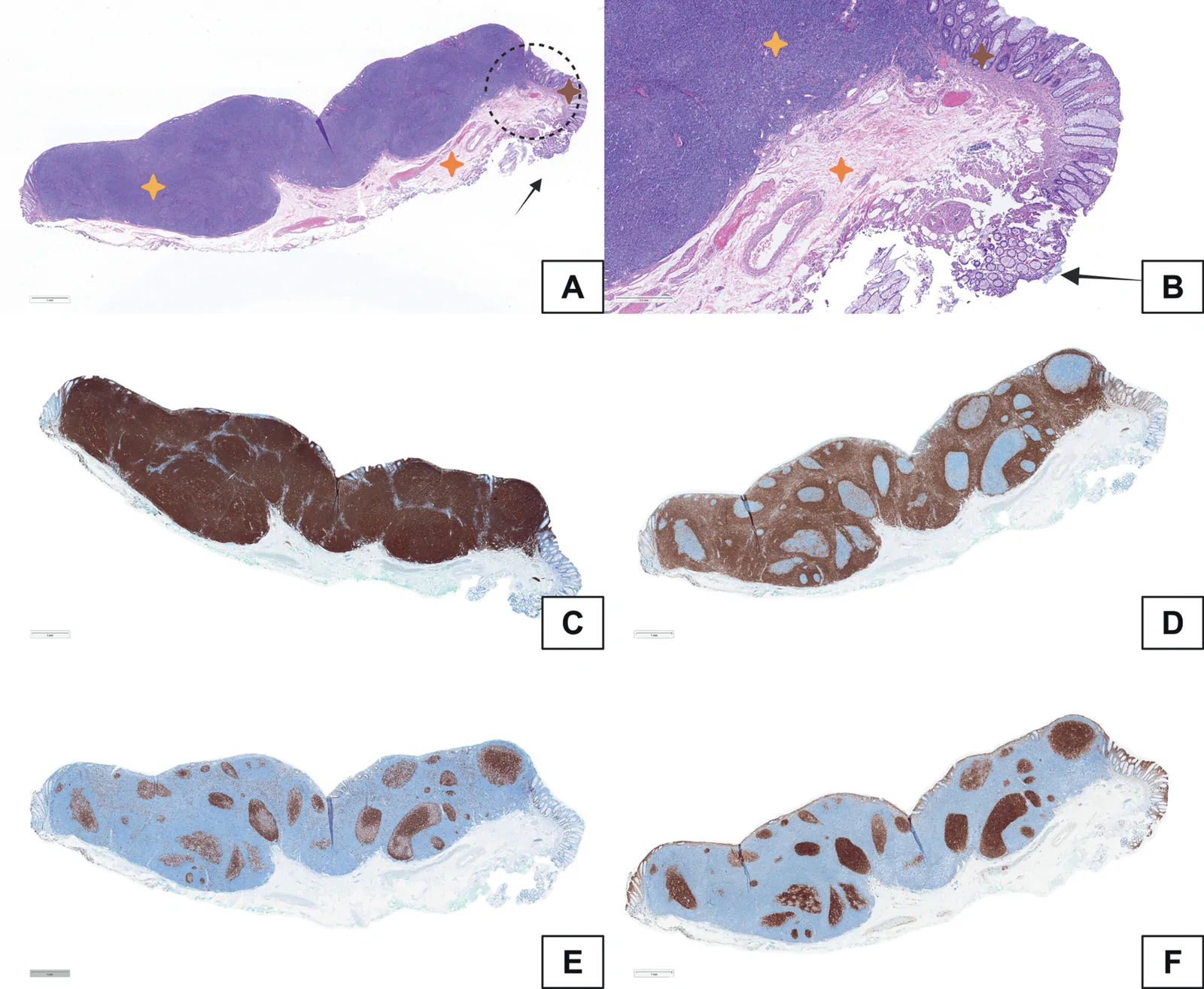
Histomorphological and immunohistochemical findings of rectal MALT lymphoma. ( **A** ) An overview showing a dense lymphomatous infiltrate expanding the lamina propria (yellow star), adjacent colorectal mucosa (brown star), and underlying submucosa (orange star). The arrow indicates the inked, electrothermally altered resection margin, which is free of lymphoma (H&E). ( **B** ) A high-magnification view of the lymphomatous infiltrate, colorectal mucosa (brown star), submucosa (orange star), and resection margin (arrow; H&E, ×20). ( **C** ) The infiltrate shows diffuse expression of the B-cell marker CD20. ( **D** ) BCL2 is expressed by the lymphoma cells, whereas the reactive germinal centers remain negative. ( **E** ) Ki-67 demonstrates the expected polarized proliferation pattern within the reactive germinal centers, whereas the proliferation index of the lymphoma is below 5%. ( **F** ) CD10 highlights the reactive germinal centers, whereas the lymphoma cells remain negative. The lymphoma cells are also negative for cyclin D1 (not shown).

Muscularis propria-level dissection should not be routine for rectal MALT lymphoma. It may be considered selectively when severe fibrosis prevents the reliable assessment of lesion depth and the deep resection boundary, after balancing the potential R0 benefit against the increased perforation risk; when an adequate more superficial plane is identifiable, this should be preferred.

Endoscopy_UCTN_Code_TTT_1AO_2AG_3AD

## Declaration of GenAI Use

Claude was only used for language editing. No generative artificial intelligence was used to create or alter any image, video or clinical data. The authors reviewed the output and take full responsibility for the final content.
